# Imaging topological and correlated insulating states in twisted monolayer-bilayer graphene

**DOI:** 10.1038/s41467-022-31851-x

**Published:** 2022-07-22

**Authors:** Si-yu Li, Zhengwen Wang, Yucheng Xue, Yingbo Wang, Shihao Zhang, Jianpeng Liu, Zheng Zhu, Kenji Watanabe, Takashi Taniguchi, Hong-jun Gao, Yuhang Jiang, Jinhai Mao

**Affiliations:** 1 School of Physical Sciences and CAS Center for Excellence in Topological Quantum Computation, University of Chinese Academy of Sciences, Beijing, 100049 China; 2 College of Materials Science and Optoelectronic Technology, Center of Materials Science and Optoelectronics Engineering, University of Chinese Academy of Sciences, Beijing, 100049 China; 3 Institute of Physics, Chinese Academy of Sciences, Beijing, 100190 China; 4 School of Physical Science and Technology, ShanghaiTech Laboratory for Topological Physics, ShanghaiTech University, Shanghai, 201210 China; 5 Kavli Institute for Theoretical Sciences and CAS Center for Excellence in Topological Quantum Computation, University of Chinese Academy of Sciences, Beijing, 100190 China; 6 Advanced Materials Laboratory, National Institute for Materials Science, Tsukuba, 305-0044 Japan

**Keywords:** Electronic properties and materials, Electronic properties and materials

## Abstract

Flat bands in Van der Waals heterostructure provide an ideal platform for unveiling emergent quantum electronic phases. One celebrated example is twisted monolayer-bilayer graphene, in which the effects of electronic correlation have been observed. Here, we report the observation via scanning tunnelling microscopy and spectroscopy of correlated insulating states in twisted monolayer-bilayer graphene, leading to the formation of an electron crystal phase. At integer fillings, the strong Coulomb interaction redistributes flat-band electrons within one moiré unit cell, producing an insulating state with vanishing density of states at the Fermi level. Moreover, our approach enables the direct visualization of an ordered lattice of topological torus-shaped states, generated by the interaction between the electron crystal and the non-trivial band topology of twisted monolayer-bilayer graphene. Our results illustrate an efficient strategy for entwining topological physics with strong electron correlation in twisted van der Waals structures.

## Abstract (web summary)

Twisted van der Waals structures represent a versatile platform to investigate topological and correlated electronic states. Here, the authors report the visualization of an electron crystal phase in twisted monolayer-bilayer graphene via scanning tunnelling microscopy, studying the coupling between strong electron correlation and nontrivial band topology.

## Introduction

Van der Waals heterostructure has been an ideal proving ground for designing and manipulating the material’s band structure^1^. After fine-tuning the lattice misalignment between the adjacent two van der Waals layers, flat bands could emerge and have been utilized to explore correlated quantum phases^2–4^. As the bandwidth of those well-isolated flat bands becomes comparable to or less than the Coulomb interaction, the strongly correlated interaction dominates the electronic behavior. In twisted bilayer graphene (tBG), this interaction-driven Mott-like insulators^2,5^, unconventional superconductivity^6,7^, and even Chern insulator^8–10^ have been realized through dedicated doping of those narrow flat bands. Extending this twisted notion to graphene multilayers would allow the flat bands to be topologically non-trivial due to the potentially reduced spatial symmetry^11–13^. Effective coupling of the band topology with those strongly correlated physics within the topological flat-bands provides an even more agile platform for exploring the emergent quantum phenomena.

Here we use scanning tunneling microscope (STM) to study the electronic properties of twisted monolayer-bilayer graphene (tMBG), where monolayer graphene is stacked on top of bilayer graphene with a slightly twisted angle. Compared with the tBG system, tMBG has a lower lattice symmetry, resulting in the topologically non-trivial flat bands near the Dirac points. The gate-tunable device configuration enables us to tune the Fermi level relative to those flat bands and leads to a transition from single-particle physics to a strongly correlated physics. With the help of STM, we can directly investigate the space-dependent spectra of those correlated states down to the nanometer scale. We observe a charge redistribution within one moiré unit cell in those correlated phases. Those redistributed charges would condense into an ordered structure and lead to an electron crystal at the integer fillings. Different from splitting the flat band in the tBG system^5,14,15^, the formation of electron crystal in tMBG at integer filling provides another way to realize the correlated insulator^16^. The electron crystal causes a spatial modulation on the local topological flat bands, which induces spatially oscillated topological properties even within one moiré unit cell. The topologically protected edge states, torus structures, are visualized directly by our spectrum imaging at those boundaries. We also provide a basic theoretical model to reproduce the electron crystal. Our result offers another avenue for realizing the correlated insulating states and combining the strong electron correlation with the non-trivial band topology for emergent quantum phenomena.

## Results and discussion

### Flat bands in monolayer-bilayer graphene

**Fig. 1:**
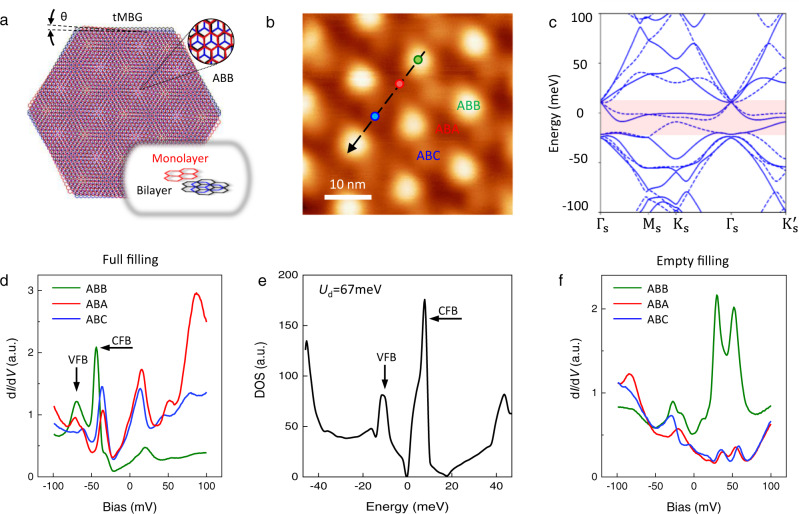
Electronic structures of the twisted monolayer-bilayer graphene (tMBG) at 1.04°. **a** A schematic atomic structure of the tMBG. *θ* is the twisted angle between the top monolayer (red honeycomb structure) and the bottom bilayer graphene (blue and black structure). **b** Scanning tunneling microscopy (STM) topography of tMBG that showing the moiré patterns at bias voltage *V*_b_ = −1 V, tunneling current *I* = 50 pA. According to the collected differential conductance (d*I*/d*V*) spectra, different stacking configurations are assigned, and ABB, ABA, and ABC label the high-symmetry regimes according to the atomic registry. The color circles indicate the sites where we collect the spectra in (**d**) and (**f**) with the corresponding color code. The dashed line indicates the trace for the spatial evolution of flat band in Fig. 2d. **c** The calculated band structures for tMBG by the continuum model. Red shadow highlights the two flat bands. Solid and dashed lines correspond to the band of **K** and −**K** valley. **d** d*I*/d*V* spectra for the three high-symmetry regimes that are taken at full-filling state (gate voltage, *V*_g_ = +40 V) (*V*_b_ = −100 mV, *I* = 200 pA). **e** The density of states (DOS) that directly deduced from the band structure in (**c**), where *U*_d_ means the vertical electrostatic potential energy drop. VFB and CFB label the valence flat band and conduction flat band in (**d**) and (**e**). **f** d*I*/d*V* spectra for the three high-symmetry regimes that are taken at empty filling state (*V*_g_ = −40 V) (*V*_b_ = −100 mV, *I* = 200 pA).

Figure 1a shows a schematic atomic structure of the tMBG sample. The moiré superstructure comprises a triangular lattice of ABB stacking (representing an on-top site stacking between the top monolayer and the bottom bilayer), alternatively surrounded by more structurally stable ABA and ABC regions^11^. The tMBG sample is scanned by STM in Fig. 1b, in which bright (dark, medium) spots correspond to ABB (ABA, ABC) stacking regions in Fig. 1a. The superlattice periodicity 13.5 ± 0.2 nm allows us to deduce the twisted angle $\theta =1.04\pm 0.02^\circ$. Its band structure is calculated by the continuum model in Fig. 1c (Supplementary Information for details, Supplementary Section I), which gives two flat bands with weak dispersion (red shadow). The d*I*/d*V* spectra (Fig. 1d) for highly electron doping (full-filling) reveal the flat bands as two prominent peaks, consistent well with the calculated density of states (Fig. 1e). Both results show that the conduction flat band (CFB) has a narrower bandwidth than the valence flat band (VFB). The wavefunctions of flat bands in Fig. 1d also show delocalization at electron doping, i.e., they have similar spectrum weight in ABB, ABA, and ABC areas. However, when we flip the backgate voltage to hole doing (empty filling), as shown in Fig. 1f, the wavefunctions of flat bands become relatively localized on the ABB part, i.e., they show higher spectral weight in the ABB area compared to the two others.

**Fig. 2:**
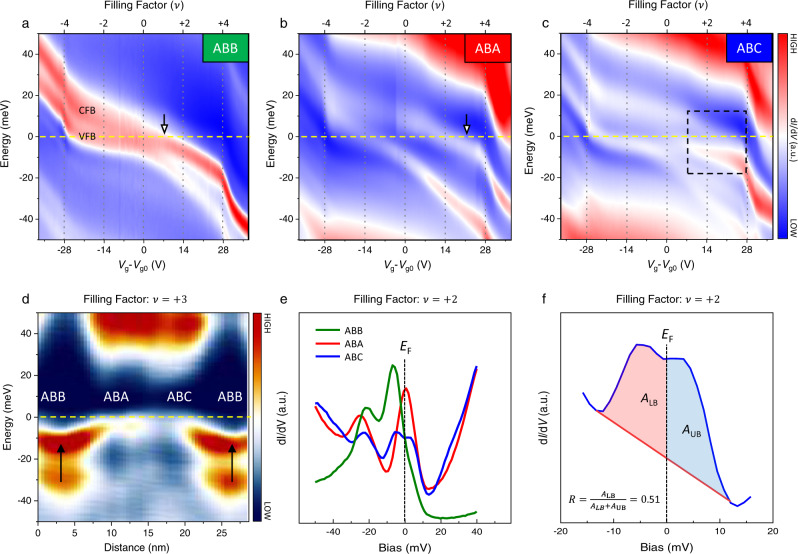
Charge separation. **a**–**c** The color contours of the d*I*/d*V* evolution as a function of doping level (*V*_g_) and filling factor ($\nu$) for ABB, ABA, and ABC, respectively (*V*_b_ = −50 mV, *I* = 200 pA), here *V*_g0_ = 3 V is the gate voltage to make the system charge neutral. The filling factors ${{{{{\rm{\nu }}}}}}$ that are deduced from the gate voltages. The yellow dashed lines in each panel indicate the Fermi level that we set as *E*_F_ = 0 meV. VFB and CFB label the valence flat band and conduction flat band in (**a**). The black arrows in **a** (**b**) label the full filing at $\nu =1$ ($\nu =3$). The dashed square in **c** highlights the correlation gap in the ABC region around Fermi level (*E*_F_). **d** Line profile of d*I*/d*V* spectra shows the flat-band bending at $\nu =3$ along the dashed line in Fig. 1b. The arrows show the electron pools in the ABB region. Dash line labels the *E*_F_. **e** d*I*/d*V* spretra at $\nu =2$ at the ABB, ABA, and ABC regions, wherein the flat bands show different filling status. Dash line labels the *E*_F_. **f** Extracting the local effective filling, *R*, of CFB from the d*I*/d*V* spectrum (blue curve) in **e**, where *A*_*LB*_ (*A*_*UB*_) is the area under the spectrum of CFB below (above) *E*_F_. By following this method discussed in the main text, the extracted *R* = 0.51, corresponding to the scenario of two electrons filling the CFB in the ABC. The dashed line indicates the *E*_F_, red line services as the base line for removing the spectrum background.

The applied backgate allows us to continuously tune the carrier doping of our tMBG from full to empty filling. We collect d*I*/d*V* spectra at ABB, ABA, and ABC regions and trace their evolution with the filling in Fig. 2a–c. For backgate voltage, *V*_g_–*V*_g0_ < −28 V (>28 V), the flat bands ultimately move above (below) the Fermi level, which allows us to define the global filling factors at *V*_g_–*V*_g0_ = −28 V (28 V) as $\nu =-4$ (${{{{{\rm{\nu }}}}}}=4$). After setting this criterion, we could deduce the global filling factor of the moiré unit cell $\nu =-4,\,-3\ldots 0,\ldots +3,\,+4$ corresponding to backgate voltage *V*_g_–*V*_g0_ = −28 V, −21 V…0, …21 V, 28 V.

### Correlated insulating states and electron crystal phase

Now we study the electronic properties of tMBG within the flat bands at partial fillings. In Fig. 2a–c, we can see an electron-hole asymmetry for the appearance of correlated behavior. There is no apparent band splitting on the hole-doped side ($\nu \, < \, 0$) in these spectra. The absence of a correlated insulating state in the hole-doping range agrees with the previous transport measurements^11,12^. While on the electron-doping side ($\nu \, > \, 0$), the gate-dependent d*I*/d*V* spectra present an intriguing mode. For the ABB and ABA stacking region (Fig. 2a, b), there is no apparent band splitting for all electron-doping ranges. Nevertheless, for the ABC region (Fig. 2c), the CFB splits into two branches with a center-to-center energy difference $\triangle E \sim 10\,{{{{{\rm{meV}}}}}}$ (dashed square in Fig. 2c and the spectrum in Fig. 2e). The emergence of this correlated state is caused by the narrower bandwidth *w*_CFB_ = 20 meV compared to the Coulomb interaction $U=\frac{{e}^{2}}{4\pi {\varepsilon }_{0}{\varepsilon }_{r}L} \sim 42\,{{{{{{\rm{meV}}}}}}}$. Here ${\varepsilon }_{0}$ (${\varepsilon }_{r}$) refers to the dielectric constant of vacuum (environment), and *L* is the superlattice periodicity of this tMBG. The $\frac{{w}_{{{{{{\rm{CFB}}}}}}}}{U} \sim \frac{1}{2}$ meets the threshold to active a correlated insulating state^17^, resulting in the band splitting in Fig. 2c. The only band splitting in ABC but absent in the other two regions suggests a spatially modulated Coulomb interaction in real space^18^.

Another noticeable feature of the strong correlation effect in tMBG is the charge redistribution within one moiré unit cell in the electron-doped regions. This is confirmed by the CFB bending down at the ABB regions in Fig. 2d. The band bending indicates that electrons prefer to fill ABB regions and form electron puddles at the partial-filling stage (Black arrows)^19–21^. To visualize this charge redistribution in real space, we first define a local filling ratio of the CFB as $R={A}_{{LB}}/({A}_{{UB}}+{A}_{{LB}})$ by following our previous method in tBG^5^ and illustrated in Fig. 2f, wherein ${A}_{{LB}}$ (${A}_{{UB}}$) is the area under the d*I*/d*V* spectrum for CFB below- (above-) *E*_F_ part (Supplementary Section IV). The value of *R* represents the local filling status of the band, e.g., *R* = 0 (1) equals locally empty (full) filling. We test it directly on the d*I*/d*V* spectrum of the ABC region at $\nu =2$, and get *R* = 0.51. This number approximates its expected value at half-filling 0.5 in the ABC region. Later, a map of *R* extracted from the spectrum imaging would bring out this filling sequence and charge redistribution in real space.

**Fig. 3:**
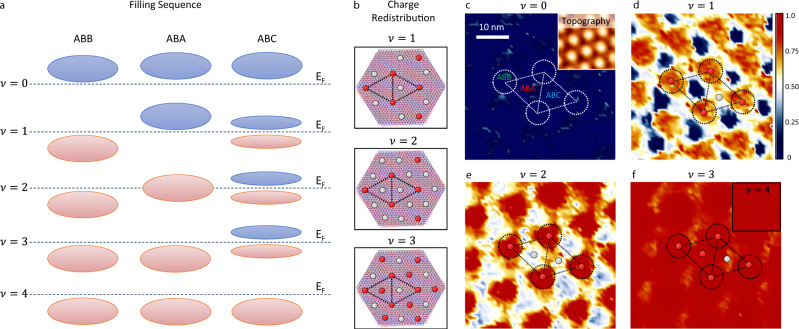
Electron crystal. **a** Schematic drawing shows filling sequences of ABB, ABA, and ABC regions as a function of global fillings $\nu$ that is listed on the left. The dashed lines indicate the *E*_F_ at each filling factor. The red (blue) ovals represent the CFB with (without) electrons occupation. **b** Summarized charge redistribution and the electron crystal from Figs. 2 and 3. Red (gray) circles superposed on three high-symmetry regions indicate the positions fully (partially) occupied by electrons. **c**–**f** Extracted local filling ratio, *R*, from every d*I*/d*V* curve in d*I*/d*V* maps for different filling factors $\nu =0$ (**c**), $\nu =1$ (**d**), $\nu =2$ (**e**), $\nu =3$ (**f**), and $\nu =4$ (inset of **f**) (*V*_b_ = −100 mV, *I* = 200 pA). The inset in **c** shows topography simultaneously obtained during d*I*/d*V* maps, which can offer the structure of the moiré superlattice and the position of three high-symmetry regions (white dotted rhombus structures in **c**–**f**). The red (gray) circles superposed on these maps represent areas with full (partial) filling, which have similar superstructures with those in (**b**). The color bar shows the local filling ratio (*R*) from 0 to 1.

The d*I*/d*V* maps at different integer fillings are collected for exploring this charge redistribution. Figure 3c–f shows the extracted contours of *R* at fixed global filling ν from 0 to 4. Initially, for $\nu =0$, there are no electrons in the whole sample, giving a uniform distribution of *R* *~* 0 everywhere (dark blue color in Fig. 3c). At $\nu =1$ (Fig. 3d), the ABB regions are the first to collect most of the electrons (labeled by the red circles in the rhombus unit cell), and the ABC regions are nearly half-filled (labeled by the gray circle). As the filling increases (Fig. 3e, f), the ABC region stays close to half-filling, and the ABA region is the second to realize complete filling (central red-ball in the unit cell in Fig. 3f). In the range of $\nu =1,\,2,\,3$, the ABC region keeps partially filled. Until the global filling factor reaches 4, the whole moiré unit cell accomplishes the flat band filling and *R* reaches 1.0 everywhere (red color in inset of Fig. 3f). This method enables us to visualize the filling sequence and electron redistribution in tMBG.

Figure 3a, b summarizes the filling sequence of the ABB, ABA, and ABC regions and electron redistribution within one moiré unit cell. At $\nu =1$, the ABB region is fully filled, while the ABA region keeps empty. A signature for CFB to entire filled is the CFB moving below *E*_F_ (marked by the black arrow in Fig. 2a). This full filling is sketched by superposing red circles on the ABB sites following the same rules in the upper panel in Fig. 3b. The ABC region realizes a half-filling featured by its band splitting and one subband sinks below *E*_F_ (Fig. 2c). We superpose the gray circles on the ABC sites to express this half-filling state in the upper panel in Fig. 3b. Following the above method, at $\nu =2$, the ABB and ABC regions keep their filling status, but the ABA regions are half-filled (Figs. 2b and 3a). So, the gray circles are added on the ABA regions (middle panel in Fig. 3b). For $\nu =3$, ABA regions become fully filled (the black arrow in Fig. 2b) and red circle is superposed in lower panel in Fig. 3b. Finally, at $\nu =4$, the ABC regions also accomplish the filling. Figure 3b shows the similar charge redistribution composed by red and gray circles compared to Fig. 3c–f.

Following the above filling sequence, we conclude a filling priority for the doped electrons among the ABB, ABA, and ABC regions. It contrasts to the delocalized flat band wavefunctions distribution in the real space in Fig. 1d. So, the electron correlation results in a transition from a uniform electron gas in single-particle physics into a localized electron superlattice at partial fillings of CFB. By following the Wigner crystal notion^16,22,23^, we define the formation of localized electron superlattice as an electron crystal at the integer fillings, and the electron correlation should play an indispensable role in this spontaneous electron crystal formation. In supplementary materials, we provide a toy model with a tripartite structure to catch this electron crystallization process. Relying on our observed different spectrum splitting behaviors among the three regions, we set three local Coulomb-interaction strengths for each sublattice site. The model qualitatively repeats the electron crystal formation (Supplementary Section III), which affirms the indispensable role of Coulomb interaction in this process. To further confirm this argument, we also examine the sample with a non-magic angle where the correlation effect could be neglected, and such an electron crystal does not emerge (Supplementary Section II and VIII).

Here we should highlight that the formation of electron crystal is the expression of correlated insulating states in tMBG. At the integer fillings, the electron crystals deplete the density of states at *E*_F_, leading to a global insulating state. For example, for $\nu =1$, the ABB region is fully filled with CFB sinking below *E*_F_, and the ABA region keeps empty with CFB sitting above *E*_F_. At the same time, the flat bands at the ABC regions prefer to split with the lower (upper) branch occupied (empty). All those three areas are insulating due to the vanishing density of states at *E*_F_, and when knitted together would make a global insulating phase (see Supplementary Section V for other fillings). This mechanism is quite different from flat-band splitting and isospin degeneracy lifting for the insulating phase in tBG^5,24–26^, or the exchange-induced global CFB splitting in twisted double bilayer graphene (tDBG)^27^. We want to emphasize that several scenarios for the metal-insulator transition have been proposed^28^, including the band splitting, electron crystals, and charge density waves. Our results directly illustrate and prove the possibility of electron crystal formation for the correlated insulating states.

This special route to realizing the correlated insulating state gives us a hint on the mechanism for the electron crystal formation. For this tMBG, it is hard to form a homogenously Mott-like insulator as the doping deviates from the one-half. But when the intersite interaction is sufficiently large, the system could roll into a charge-order phase with one area getting closer to half-filling and the other keeping empty. This electron crystal with spatially modulated charge could help realize the insulating state.

### Topologically protected superlattice

After identifying the electron crystal, we focus on its coupling with the intrinsic non-trivial band topology. Unlike the interaction-driven Chern bands in tBG^8,9^, our non-trivial band topology is inherent and derived from the band structure in a single-particle picture^29^. In our calculations, the CFB in tMBG at $\theta =1.04\pm 0.02^\circ$ has a finite Chern number $C=\pm 1$ for $\pm {{{{{\bf{K}}}}}}$ valley (Supplementary Table 1). The filling status of CFB determines the system’s topological property. As discussed above, the strong correlation induces a spatially modulated CFB filling within one moiré unit cell, where an accompanied topological property variation should be expected (Supplementary Section VII). As a result, topologically protected interface states would emerge at the boundary of two domains^30,31^. That means in tMBG system, the periodic modulation of CFB filling in real space would give rise to a periodic lattice of the topological interface states. Thus, we call it a topological order state in coordinate space.

**Fig. 4:**
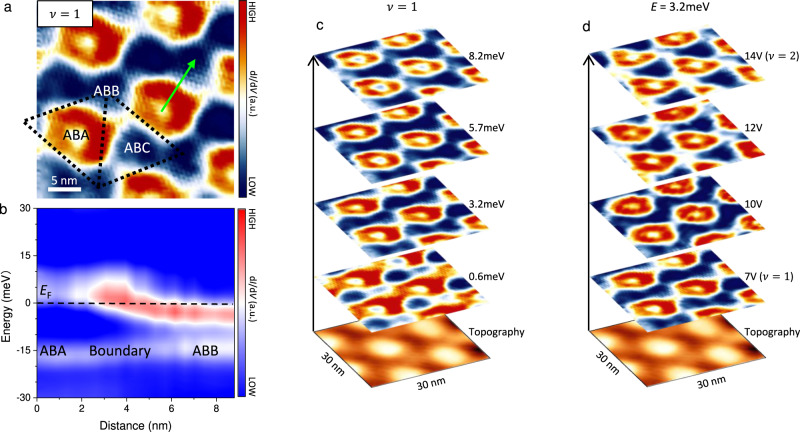
Topological torus lattice. **a** A typical d*I*/d*V* map at $\nu =1$ near *E*_F_. The torus-shaped structure shows up with bright red color (*V*_b_ = −50 mV, *I* = 200 pA). The dotted rhombus labels the moiré superlattice. **b** Evolution of the d*I*/d*V* intensity from the ABA to ABB region along the arrow in (**a**). The dashed line indicates *E*_F_. The red color near *E*_F_ indicates the enhancement of LDOS at the torus-shaped structure. **c** Stacking of d*I*/d*V* maps at $\nu =1$ as a function of energy which are labeled nearby (*V*_b_ = −50 mV, *I* = 200 pA). The bottom image shows the STM topography simultaneously obtained during these maps. **d** Stacking of d*I*/d*V* maps as a function of doping level which are labeled nearby (*V*_b_ = −50 mV, *I* = 200 pA). The bottom image shows the STM topography simultaneously obtained during these maps.

To search for those topological metallic states, we perform d*I*/d*V* maps near *E*_F_ at $\nu =1$ (Fig. 4a). At this filling stage, the CFB in ABB (ABA) regions is completely filled (unfilled). As a result, the ABB and ABA would have different topological property. Relatively, the ABB sites become topological non-trivial, and *ABA* sites act as a vacuum (see Supplementary Section XI for the ABC regions). At the boundary, an enhancement of the density of states should be anticipated due to the gapless topological states. Experimentally, we observe a succession of torus-shaped structures enclosing the ABA sites, serving as strong evidence for the anticipative topological states (Fig. 4a). The density of states along a line-cut along ABA-boundary-ABB also shows an enhanced intensity in Fig. 4b, which is in accord with the scenario of topologically protected interface states. To confirm its topological trait, we perform the same experiments on another sample without electron crystal formation and do not observe those torus-shaped structures (Supplementary Section VIII). Therefore, we can conclude that the correlation-induced electron crystal and the non-trivial band topology are crucial in forming those torus-shaped states (Supplementary Section VII). More importantly, the radius of the torus-shaped structure does not disperse with energy or doping (Fig. 4c, d). As we switch off the correlation effect by setting ${{{{{\rm{\nu }}}}}} \, > \, 4$, all those torus-shaped structures disappear, further consolidating our hypothesis on the topological torus-shaped existence (Supplementary Section VII). We also exclude other possibilities for generating the torus-shaped structures, which are discussed in Supplementary Section VI. Considering all these observations, it is legitimate to claim that this torus-shaped structure originates from the topologically protected boundary states. Combing the correlation-driven electron crystal with band topology allows for the topologically protected states and the lattice of torus-shaped structure.

## Methods

### Sample fabrication

The twisted samples are fabricated by the standard “stack and peel” method. A thin layer of PVA, poly(vinyl alcohol), film is first spin-coated on a silicon chip and baked at 90 °C for 5 min followed by spin-coating another layer of PMMA, poly(methyl methacrylate), on top and baked at 90 °C again for 5 min. Graphene layers are exfoliated on top of those PMMA/PVA thin films at room temperature, and G/PMMA/PVA layers are directly peeled off from the silicon chip by the precut tape. After the peeling, the tape with the thin film is flipped over, and graphene flakes with both monolayer and bilayer graphene connected are selected for the twisted sample stacking. The twisted mono-bilayer graphene (tMBG) heterostructure transfer process is realized by the dry-peeling-off method from the PMMA/PVA film to a pre-exfoliated boron nitride (BN) flake that sits on SiO_2_/Si surface. In order to make the electric contact for further STM characterization, Au/Ti electrodes are added by standard electron beam lithography and metal evaporation. The sample is annealed in high vacuum condition at 260 °C overnight to remove the PMMA residues. Before the final STM characterization, the sample is annealed in the ultra-high-vacuum chamber in situ to degas the surface.

### Scanning tunneling microscope characterization

The STM experiments are performed at 4.8 K. Before performing the experiments on tMBG, the system and STM tip are checked on NbSe_2_, where the CDW and superconducting gaps are used for calibration. The d*I*/d*V* spectra are collected by standard lock-in technique, with 3 mV AC modulation added on the DC sample bias.

## Supplementary information

Supplementary Information

## Data Availability

Relevant data supporting the key findings of this study are available within the article and the Supplementary Information file. All raw data generated during the current study are available from the corresponding authors upon request.
