# Supplementary material for: Imaging topological and correlated insulating states in twisted monolayer-bilayer graphene
Source: Nat Commun. 2022 Jul 22;13:4225. doi: 10.1038/s41467-022-31851-x (PMC9307793; doi:10.1038/s41467-022-31851-x)
Supplement: Supplementary file 1 — Supplementary Information [file 41467_2022_31851_MOESM1_ESM.pdf]

Supplementary Materials

Imaging topological and correlated insulating states in twisted monolayer-bilayer graphene

Si-yu Li[1,2,3,7], Zhengwen Wang[1,2,7], Yucheng Xue[1], Yingbo Wang[1], Shihao Zhang[4], Jianpeng Liu[4], Zheng Zhu[5], Kenji Watanabe[6], Takashi Taniguchi[6], Hong-jun Gao[1,3], Yuhang Jiang[2*] and Jinhai Mao[1*]

[1]School of Physical Sciences and CAS Center for Excellence in Topological Quantum Computation, University of Chinese Academy of Sciences, Beijing 100049, China

[2] College of Materials Science and Optoelectronic Technology, Center of Materials Science and Optoelectronics Engineering, University of Chinese Academy of Sciences, Beijing 100049, China

[3]Institute of Physics, Chinese Academy of Sciences, Beijing 100190, China

[4]School of Physical Science and Technology, ShanghaiTech Laboratory for Topological Physics, ShanghaiTech University, Shanghai 201210, China

[5]Kavli Institute for Theoretical Sciences and CAS Center for Excellence in Topological Quantum Computation, University of Chinese Academy of Sciences, Beijing 100190, China

[6]Advanced Materials Laboratory, National Institute for Materials Science, Tsukuba 305-0044, Japan.

Contents:

1. The non-interacting continuum model in the tMBG

2. Backgate dependent dI/dV spectra of tMBG away from magic-angle

3. Density matrix renormalization group model on repeating the cascade filling picture

4. Calculating the local filling factors from dI/dV spectra

5. Correlated insulating states by electron crystal

6. Excluding other possibilities for the torus-shaped structure in dI/dV maps

7. Gate controllable topological torus-shaped structure

8. Absence of topological torus-shaped structures without electron crystal

9. dI/dV curves under 2D maps for the ABB, ABA, and ABC regions

10. 2D maps for the ABB, ABA and ABC at another site

11. Deducing the Chern numbers for the ABC regions

References

1. The non-interacting continuum model in the tMBG

First, we describe the non-interacting physics of twisted monolayer-bilayer graphene system with the Bistritzer-MacDonald continuum model[1-5]. The Hamiltonian in this continuum model can be written as

$$H_{\mu}^{0}=\begin{pmatrix}H_{\mu,bi}^{0} & \mathbb{U}_{\mu}\\ \mathbb{U}_{\mu}^{\dagger} & H_{\mu,mo}^{0}\end{pmatrix}, \tag{1}$$

Here $H_{\mu,mo}^{0}=-\hbar v_F(k-\mathbf{K}_{mo})\cdot\sigma^{\mu}$ represents the effective Hamiltonian of monolayer graphene near the Dirac point $\mathbf{K}_{mo}$ where $\sigma^{\mu}=(\mu\sigma_x,\sigma_y)$refers to Pauli matrix, with $\mu=\mp1$ referring to the *K'/K* valley. The Hamiltonian of bilayer graphene is

$$H_{\mu,bi}^{0}=\begin{pmatrix}-\hbar v_F(k-\mathbf{K}_{bi})\cdot\sigma^{\mu} & h\\ h^{\dagger} & -\hbar v_F(k-\mathbf{K}_{bi})\cdot\sigma^{\mu}\end{pmatrix}, \tag{2}$$

The matrix h is the interlayer hopping between two aligned graphene layers

$$h=\begin{pmatrix}t_2 f(k) & t_2 f^{*}(k)\\ t_{\perp}-3t_3 & t_2 f(k)\end{pmatrix}, \tag{3}$$

In this hopping matrix, t_2= 0.21eV, $t_3\approx$ 0.05 eV, $t_{\perp}$= 0.48 eV which are extracted from the Slater-Koster hopping parameters, and $f(k)$ is the phase factor $f(k)=e^{-i\sqrt{3}ak_y/3}+e^{i(\frac{k_xa}{2}+\frac{\sqrt{3}ak_y}{6})}+e^{i(-\frac{k_xa}{2}+\frac{\sqrt{3}ak_y}{6})}$. The matrix $\mathbb{U}_{\mu}$ represents the coupling between the two neighboring layers at the twist interface. In particular, for the *K* valley with μ=-1,

$$\mathbb{U}_{\mu=-1}=\begin{pmatrix}0 & 0\\ U(\mathbf{r})e^{-i\triangle\mathbf{K}\cdot\mathbf{r}} & 0\end{pmatrix}, \tag{4}$$

Here U is the tunneling matrix between the twisted graphene bilayers

$$U(\mathrm{r})=\begin{pmatrix}u_0 g(\mathbf{r}) & u_0' g(\mathbf{r}-\mathbf{r}_{AB})\\ u_0' g(\mathbf{r}+\mathbf{r}_{AB}) & u_0 g(\mathbf{r})\end{pmatrix}, \tag{5}$$

where $\mathbf{r}_{AB}=(\frac{\sqrt{3}L_s}{3},0)$, and twist contributes to the shift between the Dirac points of the bilayer and the monolayer $\Delta\mathbf{K}=\mathbf{K}_{bi}-\mathbf{K}_{mo}$, and also lead to the phase factor $g(\mathbf{r})$ defined as $g(\mathbf{r})=\sum_{j=1}^{3}e^{i\mathbf{q}_j\cdot\mathbf{r}}$ with $\mathbf{q}_1=\left(0,\frac{4\pi}{3L_s}\right)$ where L_s is the moiré lattice constant. The intrasublattice interlayer tunneling constant u_0 (~78 meV) is always smaller than intersublattice interlayer tunneling constant u_0' (~98 meV) which originates from the effects of atomic corrugations.

In this non-interacting continuum model, we introduce the out-of-plane displacement field

D by adding an on-site energy $U_l = (l-1)U_d/3$ to the lth layer (l = 1, 2, 3) where $U_d = -eDd/\varepsilon_{BN}$ with ε_{BN} being the dielectric constant of BN and d denoting the total thickness of the twisted monolayer-bilayer graphene system. Because the out-of-plane displacement field increases with larger filling in the scanning tunneling microscopy (STM) experiment, in the calculations, the vertical electrostatic potential drop is set as 15meV, 30meV and 45meV at the 1/4, 1/2 and 3/4 filling respectively.

In the Supplementary Figure 1, we present the non-interacting energy bands under different displacement field. It's noted that the lowest conduction bands at different fillings are very flat such as the Coulomb interaction can dramatically influence the at conduction band.

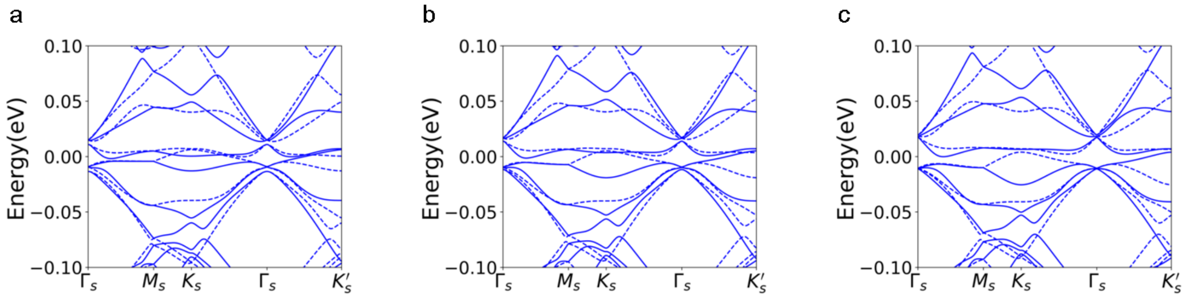

Supplementary Figure 1 The noninteracting energy bands of 1.04° twisted monolayer-bilayer graphene under U_d= 15meV field (a), U_d= 30meV field (b), and U_d= 45meV field (c).

Our calculation also shows that the flat band in tMBG at 1.04° is topological non-trivial. Both the CFB and VFB have a non-zero Chern number. Since the electron crystal happens on the electron doping part, *i.e.*, the CFB is partially filled, so we focus on the Chern number of CFB. Due to the time reversal symmetry protection, the energetically degenerated two CFB from valley K and K' owns twns the same Chern number but opposite sign. Supplementary Table 1 shows the Chern number for CFB under different displacement field.

U_d (meV)	15	30	45
**K**	1	1	1
**K'**	-1	-1	-1

Supplementary Table 1 Chern number for CFB under different U_d.

2. Backgate dependent dI/dV spectra of tMBG away from magic-angle

In the main text, we have discussed the real space carriers cascade filling and the electron crystallization triggered by the strong correlation effects. The relatively narrower bandwidth of the conduction flat band suggests that the strong correlation dominates the fundamental electronic properties of the tMBG. To further corroborate it, we also performed the same experiments on tMBG sample but with a different misalignment angle, $\theta = 0.9°$.

As shown in Supplementary Figure 2, there are two different main features in spectra at $\theta = 0.9°$ from the spectra at $1.04°$. Firstly, for the dI/dV spectra in the ABC region, there is no band splitting for both conduction or valence band for tMBG at $\theta \sim 0.9°$. This suggests that the electron-electron interaction does not dominate its electronic properties. Secondly, the gate-dependent dI/dV spectra among these three regions do not show the real space cascade filling and related electron crystallization at $\theta \sim 0.9°$. Actually, the evolution of the bands as a function of backgate synchronizes quite well among the three high-symmetry regions. It means that the single particle band model could explain those electronic structure. Considering those two aspects, we conclude that the electron crystallization is an expression of the strongly correlated physics in tMBG at $1.04°$.

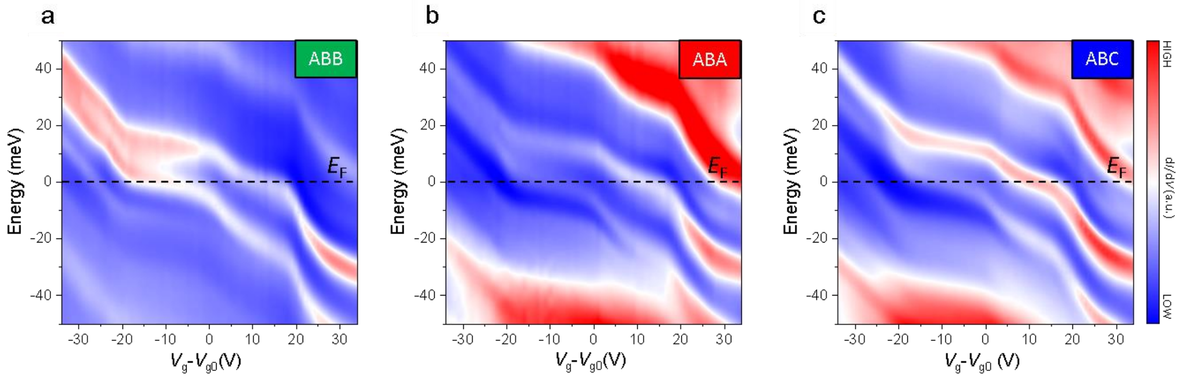

Supplementary Figure 2 Gate-dependent dI/dV spectra for three high symmetry regimes in tMBG with twisted angle $\theta \sim 0.9°$. Dashed lines label the Fermi level (E_F).

3. Density matrix renormalization group model on repeating the cascade filling picture

Here we propose a toy lattice model to simulate the cascade filling in tMBG on a tripartite lattice that comprises three triangular sublattices ABB, ABA and ABC. In this model, we assign three different local Coulomb repulsions U_{ABB}, U_{ABA} and U_{ABC} to the three areas, and consider

the nearest-neighbor hopping to capture the real space cascade filling. Our experimental results inspire the employment of the sublattice dependent Coulomb interactions: 1) Only two split Hubbard bands are observed in the ABC region, suggesting its relatively stronger Coulomb interaction than the other two regions, 2) we discover quite different LDOS behaviors at the same global filling ν in these three regions and identify the filling sequence, which might be induced by the different local interaction strength, particularly considering their same kinetic energy from the band picture, 3) the electron pools have different profile in Fig. 2d which may be created by different local potential difference. Supplementary Figure 3 shows the main results of our calculation, both the color and the radius of the colored dots are proportional to the occupation of the electrons. The model calculations do repeat a real space cascade filling among the three high-symmetry regions with the filling priority to the smallest-U area (ABB region) and last full filling to the largest-U area (ABC region), qualitatively consists with the experimental observations. We also confirm the robustness of such theoretical results over a wide range of parameters. This toy lattice model used here qualitatively captures the main features of real space cascade filling and the electron crystallization, though the mechanism of Coulomb repulsion modulation and electron crystallization may need a further theoretical study.

The Hamiltonian of the toy model on a tripartite lattice can be written as

$$H = -\sum_{\mathbf{r},\mathbf{r}',\sigma} t_{\mathbf{r}\mathbf{r}'}(c^{\dagger}_{\mathbf{r}\sigma} c_{\mathbf{r}'\sigma} + h.c.) + \sum_{\mathbf{r}} U(\mathbf{r}) n_{\mathbf{r}\uparrow} n_{\mathbf{r}\downarrow}, \tag{6}$$

Here, $\mathbf{r}$ denotes the site position in the Moiré lattice. $c^{\dagger}_{\mathbf{r}\sigma}$ ($c_{\mathbf{r}\sigma}$) and $n_{\mathbf{r}\sigma}$ represent the electron creation (annihilation) operators and number operators at site $\mathbf{r}$ with spin σ (σ = ↑, ↓), respectively. The local Coulomb repulsion $U(\mathbf{r}) \equiv U_1$ when $\mathbf{r} \in \mathrm{ABC}$, $U(\mathbf{r}) \equiv U_2$ when $\mathbf{r} \in \mathrm{ABA}$ and $U(\mathbf{r}) \equiv U_3$ when $\mathbf{r} \in \mathrm{ABB}$. In our calculation, we only consider the hoppings between the nearest neighbors and set $t_{\mathbf{r}\mathbf{r}'} \equiv t$ as the unit of energy. Here we would like to point out that this toy model may not capture all nature of tMBG due to the Wannier obstructions when writing down a tight-binding model; however, as a starting point for theoretical study, it may provide some intuitions on the main features of real space cascade filling and the electron crystallization at integer fillings.

We perform the large-scale density-matrix renormalization group (DMRG) simulations to study the sequence of filling in Hamiltonian (6). DMRG has been widely used to study various

correlated problems and proved to be one of the most powerful approaches. We consider the tripartite lattice with cylinder geometry as usually adopted by DMRG. The triangular lattice is

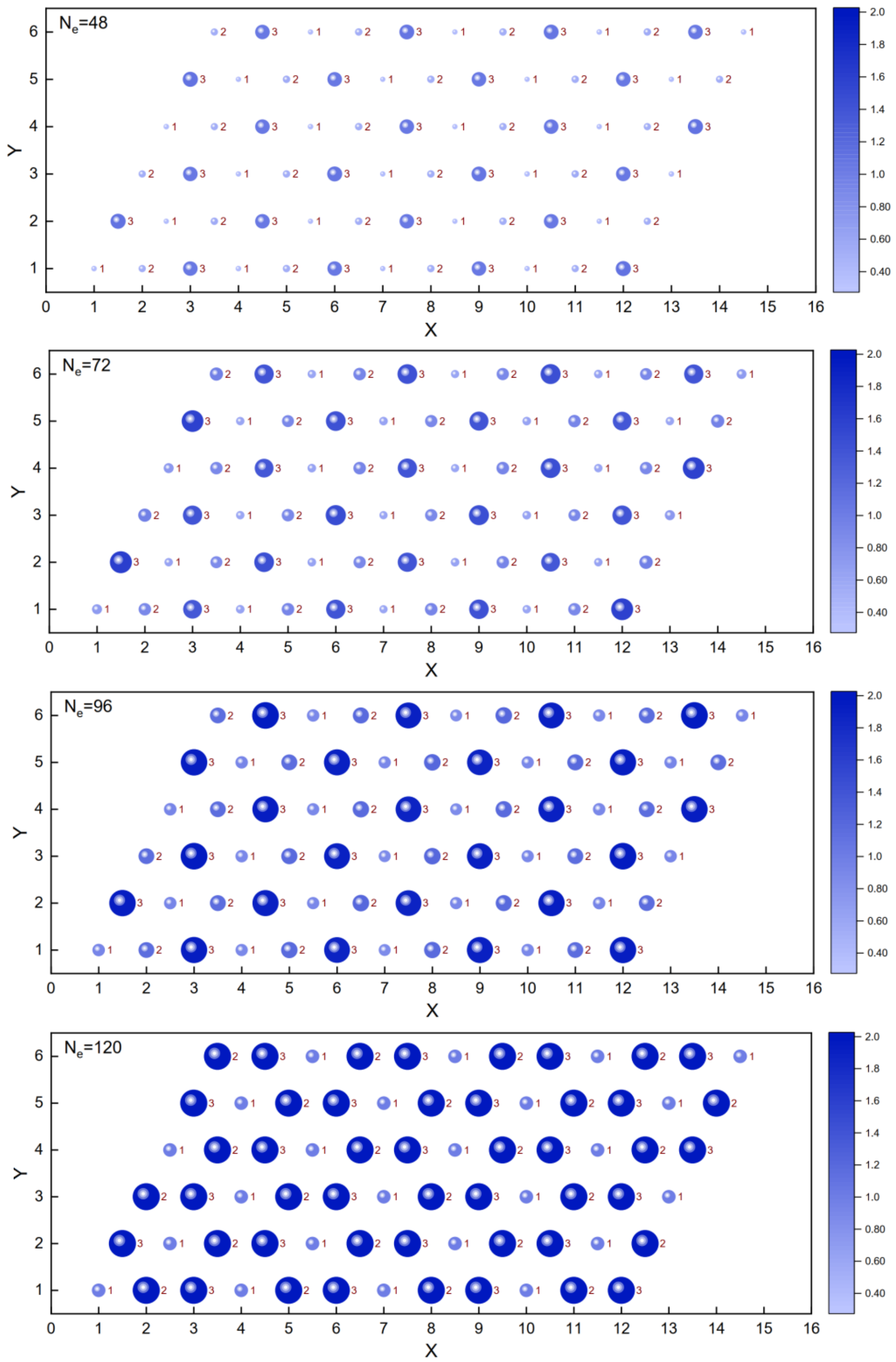

Supplementary Figure 3 DMRG results on modeling the real space cascade filling within each moiré unit cell. The Number of Ne represents the total electrons that are doped to the lattices, and the global filling factor could be calculated by $Ne/2N$ with $\mathbf{N} = \mathbf{L_x} \times \mathbf{L_y}$, here in

our model $\mathbf{N} = \mathbf{12} \times \mathbf{6}$. Both the color and size of the ball represent the local number of the electrons that occupy the sublattice.

spanned by the primitive vectors $\mathbf{e_x} = (1,0)$ and $\mathbf{e_y} = (\frac{1}{2}, \frac{\sqrt{3}}{2})$ with size $N = L_x \times L_y$.The periodical boundary condition is implemented along $\mathbf{e_y}$ direction and thus the circumference of the cylinder is L_y. It requires the integral multiple of 3 for both L_x and L_y in order to accommodate the whole period. Due to the exponential increase of the DMRG computational cost with L_y, we primarily focus on L_y = 6 cylinders with fixing the aspect ratio $\frac{L_x}{L_y} = 2$. We also have confirmed that L_y = 3 cylinders give consistent results. Here, we set the bond dimension up to *D*=50,000 ~ 60, 000. The fillings can be accurately tuned in our calculation since we have implemented various symmetries in our DMRG program such the conservation of total charge. Also, in our model, in order to exclude any boundary effect on modulating the charge distribution, we have tested different sizes $L_x \times L_y$, like 12×3,24×3,36×3, and all of them are consistent with the results presented here.

We first obtain the ground state $|\Psi_0\rangle$ of the Hamiltonian (1) and then calculate the charge density distribution $n_r = \sum_\sigma \langle \Psi_0 | c^\dagger_{\mathbf{r}\sigma} c_{\mathbf{r}\sigma} | \Psi_0 \rangle$ at different filling factors. We find our results are robust in a wide range of parameters provided that $U_1 \gg U_2 \gg U_3$. Our results shown in Supplementary Figure 3 do exhibit a real space cascade filling among the three high symmetry regions with the filling priority to the smallest U area (ABB region) and last full filling to the largest U area (ABC region), consistent with the experimental observations. Although this model qualitatively captures the main features of the cascade filling and the electron crystallization, the mechanism of Coulomb repulsion modulation and electron crystallization still calls for further theoretical study.

4. Calculating the local filling factors from d*I*/d*V* spectra

Fig. 3 in the main text shows the electron crystal configuration by plotting the spatial evolution of the local filling ratio, *R*, at four global filling factors, $\nu = 0, 1, 2, 3$. As discussed in the main text, the global filling factors could be directly determined from Fig. 2, especially for $\nu = \pm 4$, which sets the boundary of the flat bands filling. Then $\nu = 0, \pm 1, \pm 2, \pm 3$ could be automatically calculated. However, the local filling ratio is not a trivial function of doping

level, especially when putting the backgate dependent dI/dV spectrum from the three areas together (Fig. 2). By following our previous method in magic-angle twisted bilayer graphene[6], we define the local filling factor $R = {A_{LB}}/{(A_{UB} + A_{LB})}$, where $A_{LB}(A_{UB})$ is the area under the spectrum of CFB below (above) E_F (see Supplementary Figure 4).

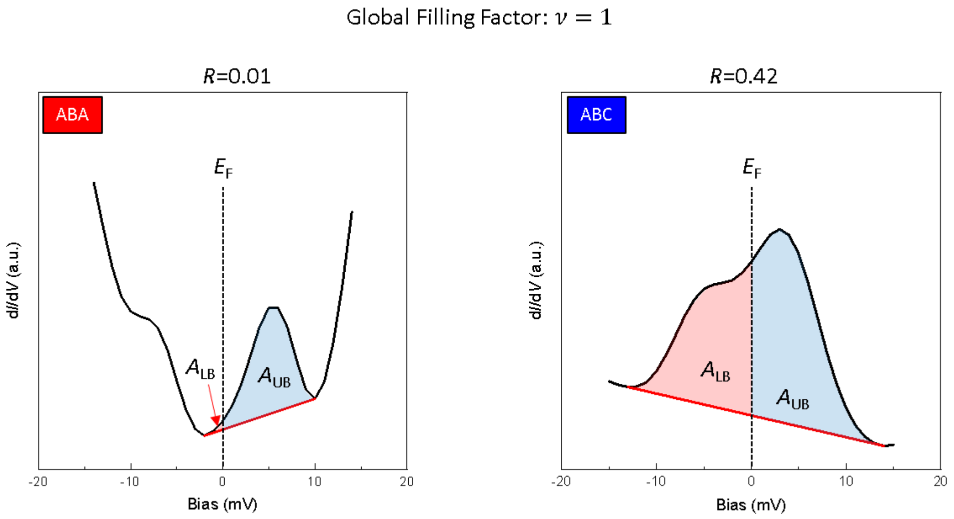

Supplementary Figure 4 Determine the local filling factor R by the area ratio below E_F from the dI/dV spectrum. More details can be found in the text. Here the global filling factor $\nu = 1$ directly reads from the applied gate voltage. The left panel is a typical dI/dV spectrum from the ABA region, and the right panel is a typical dI/dV spectrum from the ABC region.

Here are the steps for getting to those areas. Firstly, the Dirac point and top band edge of CFB are selected as the boundaries for calculating the spectrum area. Secondly, dI/dV spectrum background is subtracted to get a more accurate result. But it is worth noticing that with or without the background will not dramatically change the conclusion, since this has been tested in twisted bilayer graphene. Thirdly, A_{LB} is calculated by integrating the dI/dV spectrum intensity form the determined Dirac point to E_F. A_{UB} is calculated in a similarly way but from E_F to the top band edge of CFB. In Supplementary Figure 4, we give some examples of how we extract the local filling factors R by this method at global $\nu = 1$.

5. Correlated insulating states by electron crystal

In the main text, we have discussed how could the electron crystal interacts with the local filling of the flat band to make a correlated insulating phase. As a typical example, we discussed the $\nu = 1$ case. We provide extra details for $\nu = 2, 3, 4$ on realizing the insulating state to

complete this process, 1) for $\nu = 2$, even though the ABA region becomes partially filled and has a finite density of states at the Fermi level, the insulated ABB and ABC regions wrap the metallic ABA regions. As a result, no conducting channels are available for the electrons. 2) while for $\nu = 3$, both ABB and ABA regions are fulfilled, and the ABC regions still hold the correlated gap at Fermi level. Since there is still no available density of states at the Fermi energy, the system falls into insulating states again. 3) at the filling factor $\nu = 4$, all the bands in the three domains are occupied, and the Fermi level moves into the single-particle bandgap, making the system insulating.

6. Excluding other possibilities for the torus-shaped structure in dI/dV maps

There are also other possibilities for generating torus-shaped structure in this moiré system. 1) tip-induced local charging effect, where the torus radius should vary with the energy. We could exclude this possibility because we have a robust ring structure that does not dispersive with the energy, Fig. 4c. 2) tip bias induced band bending as in twisted transition metal dichloride, but once again, its radius needs to change with energy. 3) the intrinsic wavefunction polarization in the real space of CFB gives the ring-shaped structure. However, as we tune Vg = -40V, where the interaction effect could be ignored, we do not see those torus-shaped structure in the dI/dV maps (shown in the following session). Our results do not support those hypotheses. Taking all of those into account, our data suggests that we have the topological torus-shaped lattice in the real space.

7. Gate controllable topological torus-shaped structure

In the main text, we have observed topological torus-shaped structure near $\nu = 1$. Here we want to emphasize again that those topological torus-shaped structures only appear when the strong correlation effect induced electron crystal existing, which ensures a dramatic band filling modulation. The strong correlation effect could be switched on or off by an external electric field in the twisted heterostructures. In Supplementary Figure 5, we show the dI/dV maps near the flat band and Fermi energy at V_g-V_{g0} = -40V, wherein the global filling factor $\nu < 4$ and flat bands are empty. In this case, the correlation effect is neglectable. Either the

flat band or the electronic state near E_F does not show any torus-shaped structure, *i.e.*, the torus-shaped structure results from the strong correlation effect. We also test the dI/dV maps at different backgate, like V_g-V_{g0} = -3V (weak hole dope) and V_g-V_{g0}=14V (electron dope), Supplementary Figure 6. A direct comparison shows that the torus-shaped structures emerge only with electron crystal, *i.e.*, the Chern number modulation in real space.

By taking all those into account, we conclude that the torus-shaped structure results from the combination of nontrivial band topology and strong electron correlation. The special band structure in tMBG allows us to visualize the topological phase directly without any extra magnetic field.

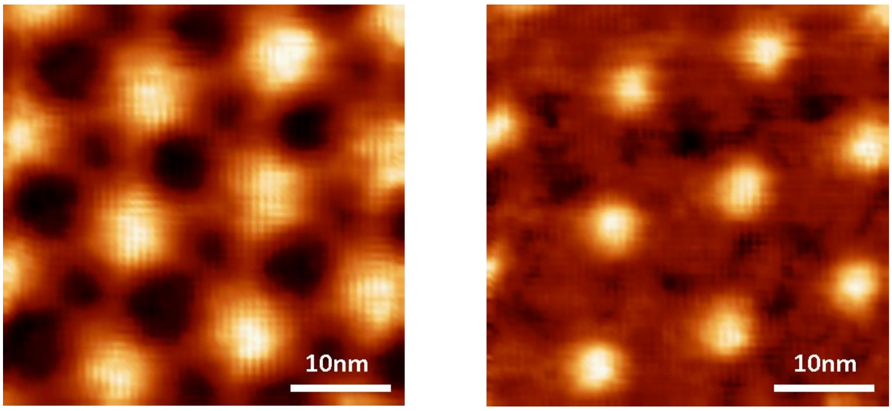

Supplementary Figure 5 dI/dV maps for the tMBG at full filling (V_g-V_{g0}= -40V). The left panel shows the dI/dV map at the flat band energy of 48.7mV, wherein the bright spots are the ABB regions. The right panel shows the dI/dV map at E_F; also the bright spots are the ABB regions.

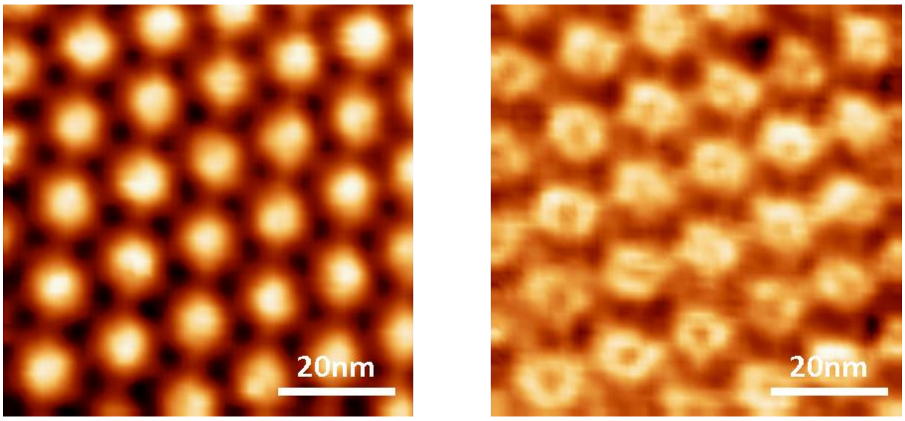

Supplementary Figure 6 dI/dV maps at 0.6mV for V_g-V_{g0} = -3V (left panel), and V_g -V_{g0} = 14V (right panel). The bright spots in the left panel represent the ABB regions. In contrast, the torus-shaped structure in right panel circulates the ABA regions.

8. Absence of topological torus-shaped structures without electron crystal

In the main text, we have reported the emergence of the topological torus-shaped structure due to the electron crystal formation. In order to confirm that those topological torus-shaped structures are electron crystal related, we have performed the same experiments in tMBG but at different twisted angles, θ~0.9°. Supplementary Figure 7 shows one of the typical dI/dV maps for tMBG without electron crystal states emerging. In the absence of the electron crystal, no torus-shaped structures are observed on the map, confirming the crucial role of the electron crystal in the topological torus-shaped structures formation.

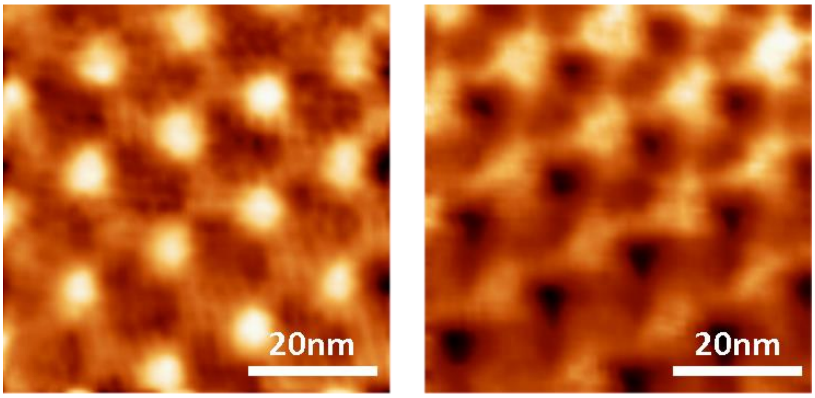

Supplementary Figure 7 Left panel: STM topography of the tMBG with θ~0.9° (V_b = -50mV, I = 100pA). Right panel: dI/dV map at the energy of 3.8mV for the same area as in the left panel with ν~1. The bright spots in the topography and dI/dV map correspond to the *ABB* regions.

9. d*I*/d*V* curves under 2D maps for the ABB, ABA, and ABC regions

In the main text, we have shown the 2D maps of ABB, ABA, and ABC regions (Fig.2 a-c). In order to show more details of these maps, we put the dI/dV curves in Supplementary Figure 8.

10. 2D maps for the ABB, ABA and ABC at another site

In the main text, we have shown one set of 2D maps of ABB, ABA, and ABC. In order to prove the reproducibility of our results, we show one extra set of 2D maps from another site in Supplementary Figure 9.

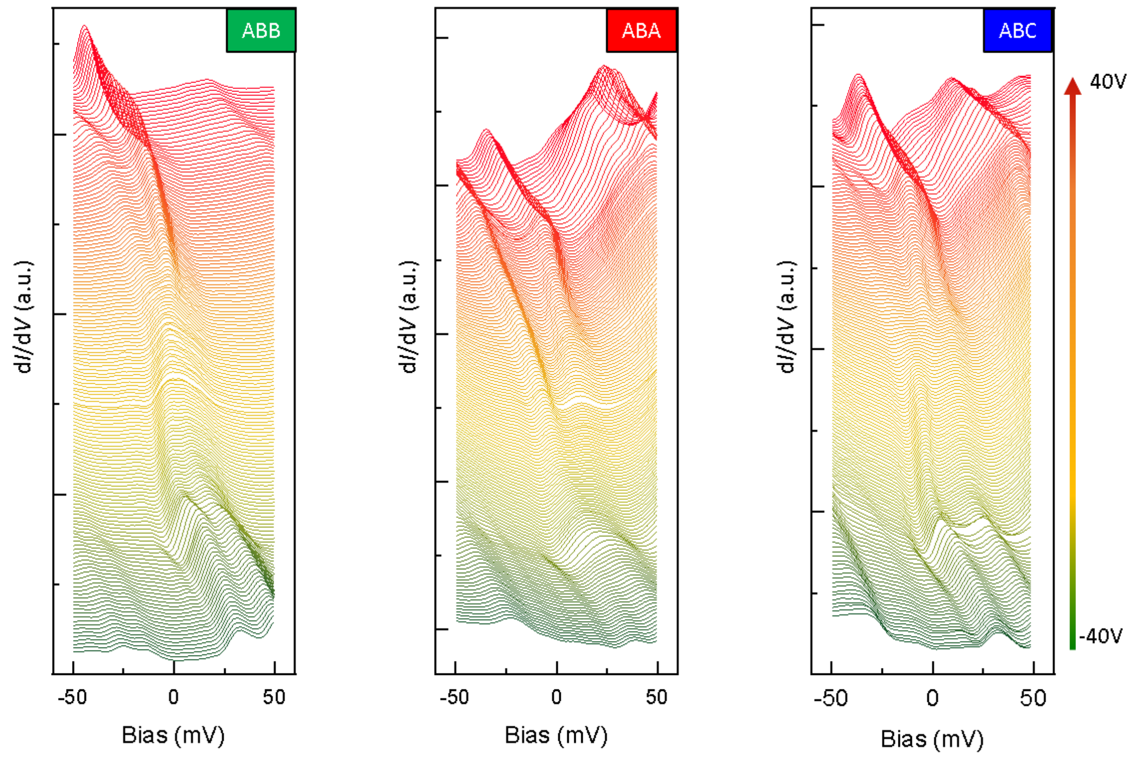

Supplementary Figure 8 the dI/dV curves under 2D maps shown in the main text.

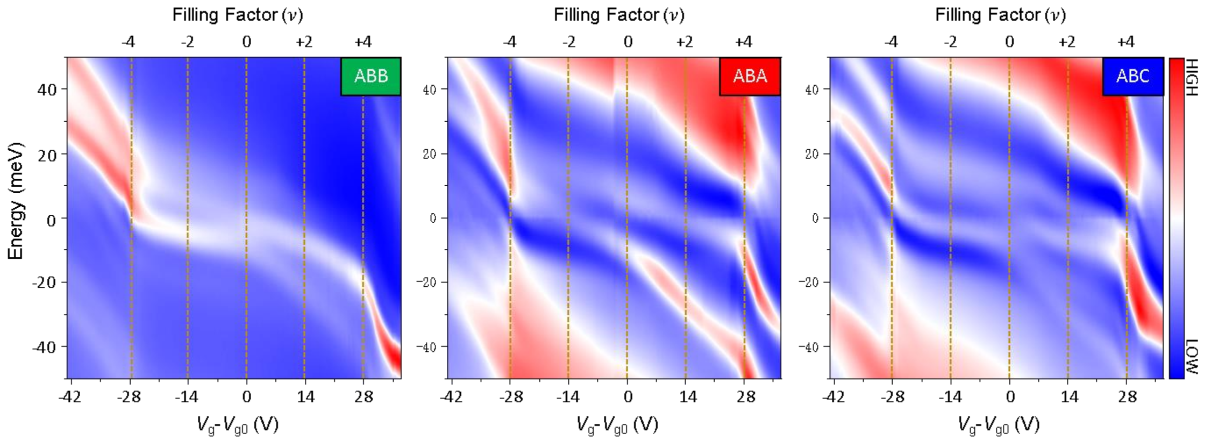

Supplementary Figure 9 2D maps for the ABB, ABA and ABC at another site (V_b = -50mV, I = 200pA).

11. Deducing the Chern numbers for the ABC regions

The torus-shaped structure further requests a topologically protected state emerging between the ABA and ABC region. This implies that the electron correlation in ABC region has to give the nonzero Chern number. For example, this could happen when the CFBs are spin split in the *ABC* region. A more quantitative theoretical modeling of such topological torus state

is beyond the scope of the present work, which we leave for future study. The band topology and strong correlation-driven topological Chern band together give the torus-shaped structure. In recent twisted graphite systems, the strong correlation has been a powerful tool for driving the non-trivial topological phase. Our results demonstrate a new vehicle on coupling the band topology and strong correlation for novel topological phase.

References

1 R. Bistritzer and A.H. MacDonald, Moiré bands in twisted double-layer graphene. *Proc. Nat. Acad. Sci.* **108**, 12233-12237 (2011).

2 Z. Ma, S. Li, Y.-W. Zheng, M.-M. Xiao, H. Jiang, J.-H. Gao, and X.C. Xie, Topological flat bands in twisted trilayer graphene. *Sci. Bull.* **66**, 18-22 (2021).

3 L. Rademaker, I. V. Protopopov, and D. A. Abanin, Topological flat bands and correlated states in twisted monolayer-bilayer graphene. *Phys. Rev. Research* **2**, 033150 (2020).

4 J. Liu, Z. Ma, J. Gao, and X. Dai, Quantum Valley Hall Effect, Orbital Magnetism, and Anomalous Hall Effect in Twisted Multilayer Graphene Systems. *Phys. Rev. X* **9**, 031021 (2019).

5 S. Zhang, X. Dai, and J. Liu, Spin polarized nematic order, quantum valley Hall states, and field-tunable topological transitions in twisted multilayer graphene systems**.** *Phys. Rev. Lett*. **128**, 026403 (2021).

6 Y. Jiang, X. Lai, K. Watanabe, T. Taniguchi, K. Haule, J. Mao and E.Y. Andrei. Charge order and broken rotational symmetry in magic-angle twisted bilayer graphene. *Nature* **573**, 91–95 (2019).
